# CuAPO-5 as a Multiphase Catalyst for Synthesis of Verbenone from α-Pinene

**DOI:** 10.3390/ma15228097

**Published:** 2022-11-16

**Authors:** Hongyun Wang, Haijun Cheng, Fang Lai, Deyuan Xiong

**Affiliations:** 1 School of Chemistry and Chemical Engineering, Guangxi University, Nanning 530004, China; 2 Guangxi Key Laboratory of Petrochemical Resource Processing and Process Intensification Technology, School of Chemistry and Chemical Engineering, Guangxi University, Nanning 530004, China

**Keywords:** CuAPO-5, α-pinene, selective oxidation, verbenone

## Abstract

Copper(II)-containing aluminum phosphate material (CuAPO-5) was synthesized hydrothermally and used as a multiphase catalyst for the oxidation of α-pinene to verbenone. The catalysts were analyzed using X-ray diffraction (XRD), Brunauer–Emmett–Teller (BET) surface area techniques, X-ray photoelectron spectroscopy (XPS), and ammonia temperature programmed reduction (NH_3_-TPD). Scanning electron microscopy (SEM), X-ray energy spectrometry (EDS), inductively coupled plasma emission spectroscopy (ICP-OES), Fourier infrared spectroscopy (FT-IR), and ultraviolet-visible spectroscopy (UV-vis) were performed to characterize the material. The effects of reaction temperature, reaction time, n(α-pinene)/n(TBHP), and solvent on the catalytic performance of CuAPO-5 were investigated. The results show that all the prepared catalysts have AFI topology and a large specific surface area. Copper is evenly distributed in the skeleton in a bivalent form. The introduction of copper increases the acid content of the catalyst. Under the optimized reaction conditions, 96.8% conversion of α-pinene and 46.4% selectivity to verbenone were achieved by CuAPO-5(0.06) molecular sieve within a reaction time of 12 h. CuAPO-5(0.06) can be recycled for five cycles without losing the conversion of α-pinene and the selectivity to verbenone.

## 1. Introduction

Turpentine is a natural product that can be obtained by distilling pine resin [1]. Turpentine can also be obtained as a by-product in the paper industry [2]. The study of turpentine and its related applications is not only of great commercial value but also of great significance to the rational use of resources. α-pinene, the main component of turpentine, is a renewable resource with high added value and low price [3,4]. For the oxidation of α-pinene, epoxidation can take place at the double bond to form α-pinene epoxide, and oxidation can take place at the allyl position to form verbenol and verbenone [5,6]. These two competing reactions are influenced by the catalyst activity and the stability of the free radicals formed during oxidation [7]. α-pinene and its oxidation products are important fine chemical intermediates. α-pinene epoxide is widely used in fragrance, cosmetics, food, and pharmaceutical industries [3]. Among them, verbenone has a high commercial value as one of the main oxidation products, such as the preparation of insecticides, the treatment of pain and inflammation, the synthesis of paclitaxel as a substrate to treat cancer, and the preparation of fragrances [8,9,10,11]. 

Verbenone can be extracted from natural plants, but the amount obtained is not enough to meet the market demand, so it is very important to use the synthetic method to prepare verbenone. According to previous reports, there are many ways to synthesize verbenone. R. Agrawal et al. improved the production of verbenone from trace (under screening conditions) to 3.28 mg/100 mL (equivalent to the mole conversion rate of substrate 16.5%) by improving the culture medium for fungi [12]. Giulia R. Gheorghita et al. used a cocktail of oxidoreductase enzymes (2-1B peroxidase and M120 laccase) to achieve optimal performance (such as 80% (+)-α-pinene conversion and 70% yield in verbenol), after 5 h of culture, under optimal conditions [13]. Although this biological method has the advantages of green environmental protection, it is sensitive to the external environment, and the cultivation cost is high. In contrast, chemical methods tend to have small investment and quick results. Copper was developed for the oxidation of α-pinene at a very early stage. Badia Ait Allal et al. used CuCl_2_ as a catalyst and TBHP (Tert-butyl Hydroperoxide) as an oxidant for the oxidation of α-pinene. There was 100% conversion of α-pinene achieved, and the selectivity of verbenone was as high as 78% [14]. Although such metallic copper salts facilitate the oxidation of allyl sites, homogeneous catalysts, such as metallic salts, lead to the generation of large amounts of experimental waste, which are difficult to dispose of and pollute the environment. Multiphase catalysts play a key role in solving the economic and environmental problems of the chemical industry with their ease of processing and separation [15]. Copper oxides have good redox exchange capacity (Cu^0^, Cu^+1^, Cu^+2^), which facilitates free radical-catalyzed oxidation reactions [16,17]. Lindokuhle S. Mdletshe et al. used spinel CuFe_2_O_4_, containing impurity phases Cu(OH)_2_ and CuO, as a catalyst and achieved 80% conversion of α-pinene, after 20 h oxidation of α-pinene with TBHP as an oxidant, as well as 76% selectivity for the combination of verbenol and verbenone [18]. This further indicates that copper remains selective for the allyl-site oxidation of α-pinene in a multiphase catalyst. In addition to metal oxides, crystalline inorganic porous materials, such as zeolites, are also important types of multiphase catalysts. Zeolites have been widely used in adsorption, catalysis, separation, ion exchange, and other chemical applications [19]. Aluminum phosphate molecular sieves, represented by AlPO_4_-5, have been widely studied. Aluminum phosphate (AlPO-n) molecular sieve is generally synthesized by hydrothermal [20], solvothermal [21], and ionothermal methods [22], and it has physical/chemical properties similar to zeolites, along with Lewis and Bronsted acids. AlPO_4_-5 is a three-dimensional electrically neutral skeleton made of alternating oxygen atoms, shared by AlO_4_^−^ and PO_4_^+^ tetrahedra, which has good structural and thermal stability. Weakly and moderately acidic sites can be made to appear in the AlPO_4_-5 framework by binding metal ions (transition metal ions and lanthanides) [23]. These active sites play a great role in the oxidation reaction. Balachandran Sundaravel et al. used PrAPO-5 molecular sieve prepared by the hydrothermal method as catalyst, molecular oxygen as oxygen, and chloroform as a solvent, thus achieving 92% conversion of α-pinene and 90.1% selectivity of lobenolides at 70 °C for a 12 h reaction [24]. When the +2 valent heteroatom is crystallographically substituted for Al, the aluminum phosphate molecular sieve skeleton produces an excess of negative charge, and the protons bound to maintain the skeleton charge balance produce significant Bronsted acid sites on the surface of the molecular sieve. When the molecular sieve is roasted, some of the Bronsted acidic sites are dehydrated and converted into Lewis acidic sites, resulting in a significant increase in the acidity of Bronsted and Lewis acids. When the amount of copper added to the skeleton is increased appropriately, the number of acidic sites also increases. Copper can be incorporated into the material by homocrystalline substitution or by loading, and it can be used as a catalyst for liquid-phase oxidation, with peroxides as oxidants, due to the presence of redox-active copper sites. 

In this paper, a simple and efficient synthesis route was reported. Aluminum phosphate (AlPO_4_-5) molecular sieve, with different Cu and Al ratios, was synthesized and characterized by different processes. It was first used to synthesize verbenone from α-pinene. Conventional routes for the synthesis of verbenone often involve the generation of α-pinene epoxide, whereas no α-pinene epoxide was generated using the present report, and the catalysts were easily separated. Therefore, we used CuAPO-5 as an environmentally friendly recyclable catalyst for the selective oxidation of α-pinene for the synthesis of verbenone. This is of industrial importance for exploring cheap and clean pathways for the oxidative synthesis of verbenone from α-pinene.

## 2. Materials and Methods

### 2.1. Materials

Chemicals used in this work include copper nitrate (Cu(NO_3_)_2_, AR, McLean Biochemical Technology Co., Shanghai, China), α-pinene (AR, Aladdin Chemical Co., Shanghai, China), aluminum isopropoxide (McLean Biochemical Technology Co.), triethylamine (AR, Aladdin Chemical Co.), phosphoric acid (H_3_PO_4_, 75%, Aladdin Chemical Co.), TBHP (70%, Aladdin Chemical Co.), chloroform (AR, Aladdin Chemical Co.), acetonitrile (AR, Aladdin Chemical Co.), ethyl acetate (AR, Aladdin Chemical Co. Ltd.), chloroform (AR, Aladdin Chemical Co., Ltd.), acetonitrile (AR, Aladdin Chemical Co., Ltd.), acetone (AR, Aladdin Chemical Co., Ltd.), ethyl acetate (AR, Aladdin Chemical Co., Ltd.), DMF (AR, Aladdin Chemical Co., Ltd.), and verbenone (AR, Aladdin Chemical Co., Ltd.).

### 2.2. Synthesis of CuAPO-5

CuAPO-5 molecular sieves were synthesized by hydrothermal crystallization of a gel with the following molar composition: xCu:1.0Al:1.35P:1.6TEA:35H_2_O. According to the ratio of Cu and Al, x = n_Cu_/n_Al_, the sample is denoted as CuAPO-5(x). Aluminum isopropoxide (10.78 g) was taken in a beaker, diluted with double distilled water (17.5 mL), and aged for 24 h. To this, 85% H_3_PO_4_ (8.64 g) was added under stirring, and the mixture was vigorously stirred for 2 h. Subsequently, Cu(NO_3_)_2_ (0.31 g taken in 3.5 mL water) was added slowly followed by stirring. After stirring vigorously for 5 min, the remaining water is added to it. After 2 h, TEA (4.64 g) was added, and it was stirred under closed conditions for 4h. The solution was transferred to an autoclave and kept at 100 °C for 2 h; then, it was heated to 180 °C for 8 h under auto-phase pressure. After the hydrothermal treatment, the autoclave was cooled to room temperature, and the obtained product was washed with distilled water several times, oven dried at 110 °C for 12 h, and finally calcined at 550 °C for 7 h. For the samples with AlPO_4_-5 metal ratios of 0.06 and 0.09, the same method was adopted, but the addition amount of Cu(NO_3_)_2_ was increased, respectively.

### 2.3. Catalyst Characterization

The prepared molecular sieve catalysts were characterized by XRD using an Ultima VI powder X-ray diffractometer manufactured by RIKEN, Japan, with a Cu target Kα source (λ = 0.15418 nm), a test voltage of 40 kV, a test current of 40 mA, a rotation range of 2θ: 5°~50°, and a scanning speed of 5°·min^−1^. 

A fully automated specific surface area and microporous physisorption analyzer (NOVA 2200e) from Quantachrome, Inc. (Boynton Beach, FL, USA) was used to perform N_2_ adsorption–desorption tests on the catalysts. The samples were dried and degassed at 300 °C before the N_2_ adsorption tests were performed at −196 °C. The specific surface area was calculated using the BET (Brunauer–Emmett–Teller) equation. The pore size distribution was calculated by the density functional theory (DFT) method for isothermal desorption, and the average pore size was also calculated by the density functional theory (DFT) method.

A 720ES Inductively Coupled Plasma Emission Spectrometer from Agilent, Santa Clara, CA, USA, was used to characterize the catalyst for elemental content analysis.

The F16502 Scanning Electron Microscope (Surface Microscopy Tester) from PHENOM, Rotterdam, The Netherlands, was used to characterize the surface micromorphology of the catalyst. The samples need to be gold sprayed before observation.

A Smartedx EDS spectrometer from Carl Zeiss AG, Oberkochen, Germany, was used to characterize the distribution of the elements contained in the catalyst.

The surface acid strength of the catalyst was tested using an AMI-300lite adsorption and desorption instrument manufactured by Altamira, Mclean, VA, USA.

The intelligent FTIR spectrometer can be used to study the molecular structure, as well as to check and identify organic compounds. The prepared samples were tested and characterized on a 1310-Nicolet IS50 model infrared spectrometer from Thermo Fisher Scientific, China, in the scanning wave number range of 4000–400 cm^−1^. The samples were ground with high purity KBr crystals and then mixed and pressed with the samples in a mixing mass ratio of the sample: KBr = 1:200.

A Shimadzu UV-Visible NIR spectrophotometer (UV-3600i Plus) was used to analyze the reflectance absorption curve spectrum of the catalyst. Before the test, the sample was evenly spread on the analytically pure barium sulfate substrate and compacted with a press, a scanning wavelength range of 200nm~800nm, and a scanning speed of 200 nm·min^−1^.

An X-ray photoelectron spectrometer (ThermoFischer, ESCALAB 250Xi, Waltham, MA, USA) was used for the tests. In this case, the vacuum of the analysis chamber was 4 × l0^−9^ mbar, the excitation source was Al ka-rays (hv = 1486.6 eV), the operating voltage was 14.6 kV, the filament current was 13.5 mA, and the signal was accumulated for 20 cycles. The test through-energy (Passing-Energy) was 20 ev in the steps of 0.1 eV, and charge correction was performed with C1s = 284.8 eV binding energy as the energy standard.

### 2.4. Catalyst Activity Tests

The catalyst (30 mg), α-pinene (1 mmol), and chloroform (10 mL) were taken in a 50 mL round-bottom (RB) flask equipped with a magnetic stirrer and reflux condenser. The reaction temperature was controlled at 85 °C by immersing the reaction mixture solution inside a hot oil bath. After the reaction solution temperature of 85 °C has been stabilized, 70% aq. TBHP oxidant (3 mmol) was then added, and immediately, the start of the reaction time was recorded. The reaction mixture, which has been filtered to remove the catalyst, is poured into cold water and extracted with ether. At the same time, acetonitrile washing catalyst was used. After washing the catalyst, the cleaning solution and the ether layer are combined and dried using anhydrous sodium sulfate. The solvent was removed by evaporation, and the product was analyzed by gas chromatography with the external standard method. Standard curves of α-pinene, verbenone, and verbenol, with reference to peak area (A, mV·s) and concentration (c, mg·ml^−1^), were established by gas chromatography:

A_α-pinene_ = 6.13 × 10^5^c_1_ − 8.58 × 10^4^, R^2^ = 0.9997 (1)

A_verbenone_ = 5.03 × 10^5^c_2_ − 1.75 × 10^5^, R^2^ = 0.9991 (2)

A_verbenol_ = 3.85 × 10^8^c_3_ − 4.25 × 10^4^, R^2^ = 0.9990 (3)

The composition of the α-pinene oxidation products mixture was analyzed by Shimadzu GC (Chemetrix, Kanagawa, Japan), and it was equipped with a flame ionization detector (FID) and a capillary column (using an Rtx@-Wax, 30 m × 0.25 mm × 0.25 µm). The injection temperature was 200 °C, and FID was 300 °C. The separation column oven temperature was from 70 °C to 200 °C, at a heating ramp rate of 10 °C/min, followed by the isothermal hold at 200 °C for 5 min. 

By quantitative analysis of the mixture, we can calculate the conversion rate (X, %) of α-pinene, as well as the yield (Y, %) and selectivity (Z, %) of the reaction products, such as verbenone and verbenol.

(4) $$X\%=\frac{\mathrm{The}\;\mathrm{mass}\;\mathrm{of}\;\mathrm{the}\;\alpha -\mathrm{pinene}\;\mathrm{being}\;\mathrm{converted}}{\mathrm{initial}\;\mathrm{mass}\;\mathrm{of}\;\alpha -\mathrm{pinene}}\times 100$$

(5) $$\mathrm{Yi}\%=\frac{\mathrm{The}\;\mathrm{mass}\;\mathrm{of}\;\mathrm{the}\;\mathrm{product}\;i\;\mathrm{that}\;\mathrm{is}\;\mathrm{converted}\;\mathrm{to}\;\alpha -\mathrm{pinene}}{\mathrm{initial}\;\mathrm{mass}\;\mathrm{of}\;\alpha -\mathrm{pinene}}\times 100$$

(6) $$\mathrm{Zi}\%=\frac{\mathrm{The}\;\mathrm{mass}\;\mathrm{of}\;\mathrm{the}\;\mathrm{product}\;i\;\mathrm{that}\;\mathrm{is}\;\mathrm{converted}\;\mathrm{to}\;\alpha -\mathrm{pinene}}{\mathrm{The}\;\mathrm{mass}\;\mathrm{of}\;\mathrm{all}\;\mathrm{the}\;\alpha -\mathrm{pinenes}\;\mathrm{converted}\;\mathrm{into}\;\mathrm{products}}\times 100$$

## 3. Results

### 3.1. X-ray Diffraction Analysis 

**Figure 1:**
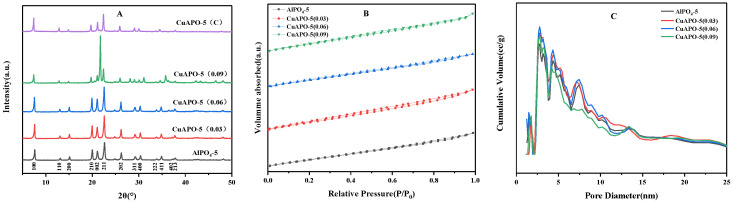
(**A**): XRD spectra, (**B**): N_2_ adsorption–desorption isotherms, (**C**): DFT pore size distribution of AlPO_4_-5 and CuAPO-5 catalysts.

To identify the crystalline phase present in the Cu-loaded AlPO_4_-5 material, the XRD analysis was carried out on CuAPO-5. Powder XRD data were assembled in the range of 5–50 degrees of 2θ. The XRD patterns (Figure 1A) of CuAPO-5 molecular sieve samples and the diffraction peaks of APO-5 standard cards coincide at the 100, 110, 200, 210, 002, 211, 202, 311, 400, 222, 411, 402, and 213 crystallographic planes [25]. It indicates that the original aluminum phosphate molecular sieve skeleton is not destroyed after the metal heteroatom completes partial lattice substitution, but it still has the AFI structure (AFI is a typical topology of AlPO_4_-5.) with no impurity signal in the diffractogram. The crystallinity of the molecular sieve decreased, accompanied by the appearance of heterocrystals when we increase the Cu/Al ratio from 0.03 to 0.09, indicating that the synthetic gel is less susceptible to doping into the APO-5 backbone when more metal salts are present. The AFI phase was retained after calcination. The relative intensity of the 110 reflection increases after calcination, while the 200 and 210 reflections decrease in intensity relative to the 100 reflection. This situation may be a relaxation of lattice distortion caused by the organic templates in the intracrystalline pore system [26]. The cell parameters of CuAPO-5 are a = b = 1.360 nm and c = 0.843 nm for a Cu/Al ratio of 0.03, and they are a = b = 1.362 nm and c = 0.844 nm for a Cu/Al ratio of 0.06. The cell parameters of CuAPO-5 are a = b = 1.372 nm and c = 0.842 nm for a Cu/Al ratio of 0.09. A slight increase in cellular parameters was observed for APO-5 loaded with a Cu/Al ratio of 0.03 and a Cu/Al ratio of 0.06 compared to APO-5. This indicates that, in the Al(III) phosphate skeleton, Al(III) is replaced by Cu(II) homocrystals (since the Cu-O bond length of 1.93 Å is significantly larger than that of 1.62 Å for Al-O).

### 3.2. ICP-OES Analysis

The copper loading in CuAPO-5 added with different Cu/Al ratios was examined using ICP-OES, and the results are shown in Table 1. ICP-OES analysis showed that the copper loading in CuAPO-5 molecular sieve, added with a Cu/Al ratio of 0.03, was 0.86 wt%. When the Cu/Al ratio was 0.06, the copper loading in CuAPO-5 was 1.88 wt%. For CuAPO-5, the addition of Cu/Al ratio of 0.09 in the synthesis gel resulted in a copper loading of 3.20 wt%. According to the results of ICP-OES, it can be concluded that the copper added at the Cu/Al ratio of 0.09 is not lost, but it is formed in other forms.

### 3.3. N_2_ Sorption Studies

It is very important to study the porosity and BET-specific surface area of AlPO_4_-5-based materials doped with Cu. The N_2_ adsorption–desorption isotherms of CuAPO-5 catalyst are shown in Figure 1B.The information on the specific surface area and pore volume determination of the samples is shown in Table 1. The low temperature N_2_ adsorption and desorption curves of each sample are type IV, which is a typical mesoporous structure, and the hysteresis loop is type H3, suggesting that these samples are elongated porous materials. 

If copper atoms do not enter the skeleton but are deposited on the outer surface of the molecular sieve, a reduction in pore volume and specific surface area is caused. The pore size distribution, plotted by the DFT method and the structural properties table of the catalysts, is shown in Figure 1 and Table 1, respectively. The pore sizes of the samples are concentrated around 2.7 nm, and the specific surface areas are concentrated around 357 m^2^·g^−1^. The specific surface area and pore volume sizes of Cu/Al = 0.03 and 0.06, are close to those of AlPO_4_-5 without copper doping, indicating that none of the three samples had copper species deposited on the outer surface of the molecular sieve and did not block the pore channels of the molecular sieve, and the copper atoms probably entered the skeleton. In addition, the samples exhibited a large specific surface area, which facilitated the uniform dispersion of the copper active centers in the AlPO_4_-5 framework. The Cu/Al ratio of CuAPO-5 molecular sieve has a slightly reduced specific surface area, which is probably because a small amount of copper enters the skeleton, and most copper is deposited on the surface.

### 3.4. Electron Microscopic Analyses

**Figure 2:**
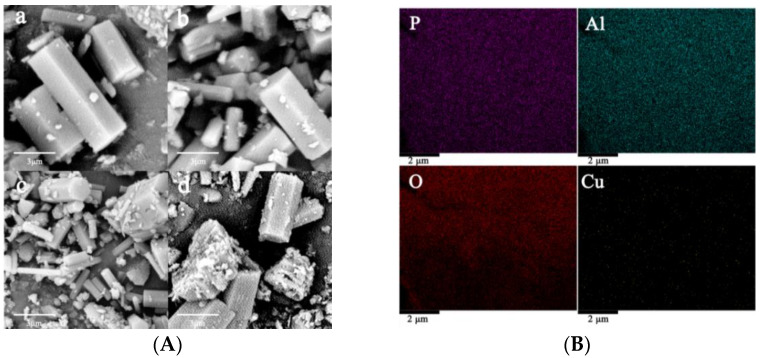
SEM images of a: AlPO_4_-5, b: CuAPO-5 (0.03), c: CuAPO-5 (0.06), d: CuAPO-5 (0.09) (**A**), and Electron mapping of CuAPO-5 (0.06) (**B**).

The morphologies of AlPO_4_-5 and CuAPO-5 molecular sieves were investigated by scanning electron microscopy, as shown in Figure 2A. Both AlPO_4_-5 and CuAPO-5 molecular sieves are hexagonal in shape, which is typical of AlPO_4_-5 molecular sieves [27]. The particle size is about 2 μm × 2 μm × 10 μm, and the surface is smooth, which indicates that the introduction of copper did not cause damage to the basic structure of AlPO_4_-5. When the Cu/Al ratio was introduced at 0.09, impurities other than hexagonal were generated, which is consistent with the appearance of heterocrystals in the XRD results. The metal signals of the CuAPO-5 molecular sieve, with a Cu/Al ratio of 0.06, were tested using the EDX point mapping technique, and the results are shown in Figure 2B. The signals of all metals appear in the EDX spectra with high dispersion.

### 3.5. X-ray Photoelectron Spectroscopy

**Figure 3:**
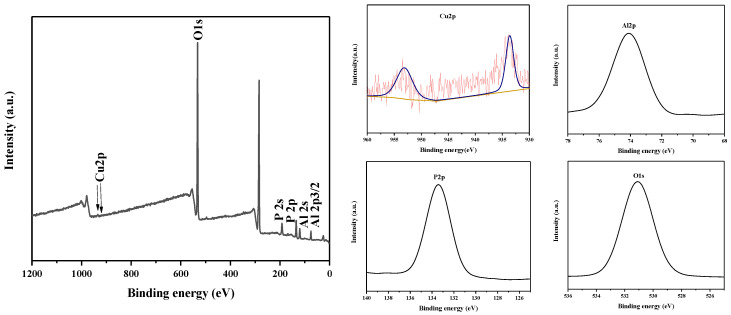
XPS spectrum of calcined CuAPO-5 (0.06).

The near-range XPS spectra of Al2p, O1s, P2p, and Cu2p, as well as the full-range XPS measurement spectrum of CuAPO-5, are shown in Figure 3, indicating the presence of O, Al, P, and Cu in the CuAPO-5 material. By XPS analysis of CuAPO-5, the oxidation state and binding energy of copper atoms in AlPO_4_-5 framework were obtained. In Figure 3, the XPS survey spectrum of Cu 2p is shown. According to the literature, the Cu 2p1/2 and Cu 2p3/2 signals located around 952.2 and 932.6 eV, respectively, revealed that Cu is in its divalent [28]. 

### 3.6. UV-Vis Spectroscopic Analysis

**Figure 4:**
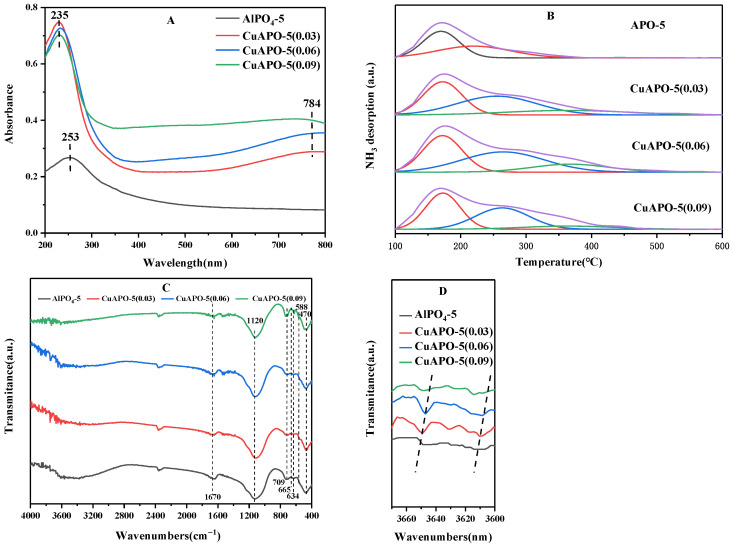
UV-Vis spectra (**A**), NH_3_-TPD curves (**B**), and FTIR spectra (**C**,**D**) of AlPO_4_-5 and CuAPO-5.

The UV−vis diffuse reflectance spectra of the roasted synthetic samples, AlPO_4_-5 and CuAPO-5, respectively, are shown in Figure 4A. As can be seen from APO-5, a broad peak appears at 253 nm, which can be interpreted as a common absorption peak of Al^3+^ (237 nm) and P^5+^ (273 nm) in the skeleton and indicates that the skeleton is a tetrahedral coordination consisting of Al^3+^, P^5+^, and O^2−^. The roasted sample of CuAPO-5 (b) shows a high intensity absorption peak at 235 nm and a medium intensity absorption peak at 784 nm, respectively, with the former being a single charge jump between Cu^2+^ and O^2−^ in the skeleton in the UV region [29,30] and the latter being a d-d jump of Cu^2+^ outside the skeleton [29]. This indicates that most of the Cu in CuAPO-5 enters the skeleton and forms [CuO_4_] tetrahedral complexes, and a small fraction exists outside the skeleton as a Cu^2+^ compound. It can be seen from the UV−vis spectrogram that the intensity of the band peak at 235 nm is significantly enhanced with increasing copper content. Combined with the XRD results in Figure 1, it can be concluded that Cu underwent homocrystalline substitution in the framework of AlPO_4_-5. In addition, the band peak intensity of the d-d transition band also increases significantly with the increase in copper content, which indicates that, in addition to homocrystalline substitution, a small amount of copper is indeed bound to the molecular sieve surface in the form of complexes.

### 3.7. Characterization of Acid Sites

**Figure 5:**
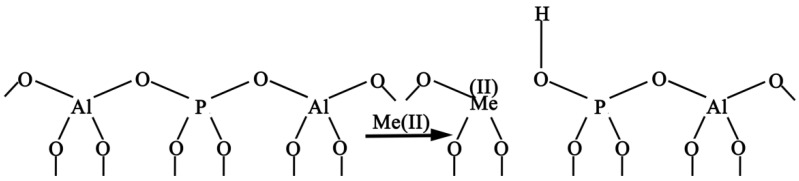
A model to explain the acidity generated by isomorphic substitution [30]. The Bronsted–Lewis interaction is evident.

NH_3_-TPD is an effective tool to verify the incorporation of heteroatoms into the AlPO_4_-5 backbone. Substitution of Al(III) or P(V) with metal ions leads to the formation of Bronsted acidic sites [31]. This is because, when divalent metals are added to the framework by homocrystalline substitution, a hydroxyl group is created to ensure charge balance [32]. The process is shown in Figure 5.

The NH_3_-TPD results of AlPO_4_-5 and CuAPO-5 are portrayed in Figure 4B and Table 1. Molecular sieves desorbed ammonia at about 170 °C, indicating the presence of weak acid sites in these molecular sieves. The ammonia desorption at around 255 °C is caused by the moderate acid site. The ammonia desorption at around 365 °C is caused by strong acid sites. For the AlPO_4_-5 molecular sieve without the addition of copper precursors, the acidic sites are mainly concentrated in the weak acid sites. Acid content of CuAPO-5 molecular sieve was significantly greater than that of AlPO_4_-5 molecular sieve after the addition of copper. The acid content of the added Cu/Al ratio of 0.06 and 0.09 was greater than that of the Cu/Al ratio of 0.03. The acid content of Cu/Al ratio of 0.09 did not increase compared to CuAPO-5 molecular sieve with Cu/Al ratio of 0.06. It is possible that, when a higher amount of Cu is introduced, it tends to develop in the direction of heterocrystals and not exactly as expected. More homocrystalline substitution occurs in the AlPO_4_-5 framework.

### 3.8. FT-IR Spectroscopy

The FT-IR can be used to detect whether the heteroatoms enter into the skeletal structure from various aspects, and the FT-IR spectra of the calcined AlPO_4_-5 and CuAPO-5 are shown in Figure 4C,D. The IR spectra of CuAPO-5 and AlPO_4_-5 are basically the same. Among them, the strong broadband at 2500~4000 cm^−1^ and the absorption peaks at 1670 cm^−1^ are attributed to the -OH stretching vibration and bending vibration of water, respectively [33]. In the FT-IR spectra of AlPO_4_-5 and CuAPO-5, no vibrational peaks of CH_2_ were found below 3000 cm^−1^, which can indicate that the template has been completely removed after roasting [33]. The strong band at around 1120 cm^−1^ is assigned to the asymmetric P-O-Al stretching vibration characteristics [26]. In addition, the energy bands at 709 and 665 cm^−1^ are P-O-Al symmetric stretching and bending vibration modes, respectively, and the bands at 634 and 588 cm^−1^ are designated as double-loop vibration modes, while the band at 470 cm^−1^ can be designated as internal T-O bending mode vibration [34]. Compared with the AlPO_4_-5 molecular sieve, CuAPO-5 produced new hydroxyl absorption peaks at 3655 cm^−1^ and 3610 cm^−1^ and increased with the increase in Cu content. It is possible that this is due to the bridging hydroxyl group Cu-OH-P produced by Cu substitution of Al [35]. When the Cu/Al ratio was 0.09, no stronger absorption peaks appeared, which indicates that the addition of excess Cu to the gel does not favor the introduction of the framework.

### 3.9. Catalytic Activity

The liquid-phase selective oxidation of α-pinene was carried out under different reaction conditions using CuAPO-5 as the catalyst. The reaction was controlled to find out the role of TBHP and the catalyst. The oxidation experiments of α-pinene and TBHP were carried out at 85 °C, under atmospheric conditions, with chloroform as the solvent. The results are shown in Table 2, indicating that when no CuAPO-5 catalyst was added, α-pinene hardly reacted and no verbenone or verbenol were produced. Similarly, in the case of the addition of CuAPO-5 catalyst, but without the addition of TBHP, the reaction did not proceed. This indicates that the reaction is the result of the interaction between CuAPO-5 and TBHP. 

In order to verify the effect of adding different amounts of Cu to AlPO_4_-5 on the oxidative synthesis of verbenone from α-pinene, experiments were carried out using CuAPO-5 molecular sieves with different Cu additions as catalysts. The reaction was carried out at atmospheric pressure and at a temperature of 85 °C, with chloroform as the solvent and TBHP as the oxidant, with the addition of a CuAPO-5 catalyst. According to the data in Table 2, the highest conversion of α-pinene and the highest selectivity of verbenone were obtained when the Cu/Al ratio of CuAPO-5 was 0.06, and they were 83.2% and 36.4%, respectively. This may be due to the fact that the largest amount of copper entered the AlPO_4_-5 framework at a Cu/Al ratio of 0.06, thus producing a large number of accessible active sites on the surface. When CuAPO-5 molecular sieve with a Cu/Al ratio of 0.03 was selected as the catalyst, the conversion of α-pinene was 81.4%, and the selectivity of verbenone was 30.6%. This may be due to the relatively small number of active sites in CuAPO-5 when the Cu/Al ratio was 0.03. When CuAPO-5 molecular sieve with a Cu/Al ratio of 0.09 was selected as the catalyst, the α-pinene conversion was 75.0%, and the selectivity of verbenone was 25.5%. It is possible that this is because the copper did not increase in the skeleton due to the increase in the amount of copper added to the gel, making the active sites not increase as expected.

**Scheme 1:**
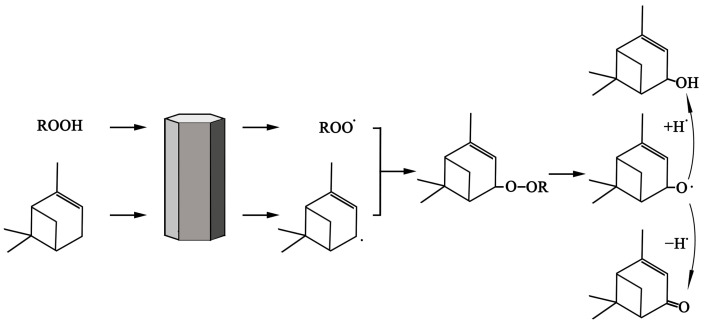
The rational reaction route of oxidation of pinene with tert−butyl hydrogen peroxide to verbenol and verbenone.

The mechanism of the catalytic oxidation reaction was hypothesized based on the experimental results and available literature reports. Cu(II) reacts with tert-butyl hydroperoxide and converts to Cu(I) to produce alkoxyl radicals and alkyl peroxyl radicals [18,29]. Alkoxy radicals produce allyl radicals after extracting allyl hydrogen from α-pinene [36]. Alkyl peroxy radicals react with allyl radicals, and then, the products decompose to verbenol and verbenone [18,29,37]. The preliminary mechanism of α-pinene oxidation, for the synthesis of verbenone and verbenol, is shown in Scheme 1.

### 3.10. Effect of Different Reaction Parameters on α-Pinene Oxidation Rates

After activity screening of the catalyst samples, CuAPO-5(0.06) was used as the best catalyst to further evaluate the effects of temperature, time, n(TBHP):n(α-pinene), and solvent variations on it, as well as to test its stability.

#### 3.10.1. Effect of Reaction Temperature

**Figure 6:**
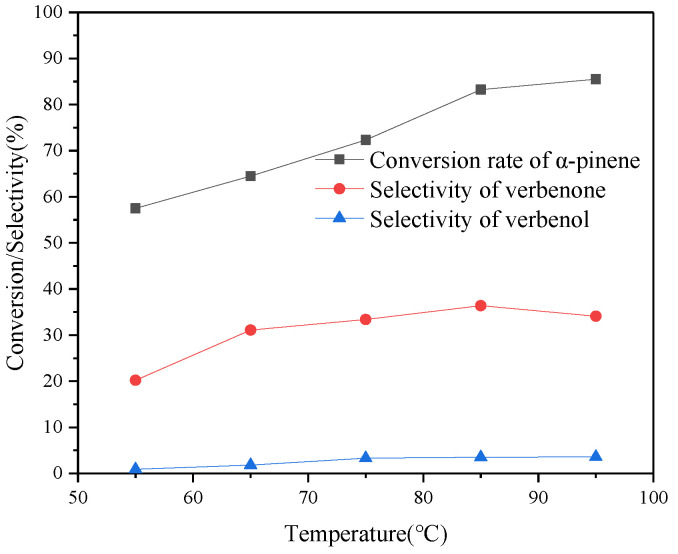
Variation of temperature in the oxidative synthesis of verbenone by α-pinene and TBHP. Reaction conditions: CuAPO-5(0.06) 100 mg, α-pinene 1 mmol, TBHP 2 mmol, TCM 10 mL, reaction time 10 h.

The oxidation of α-pinene was carried out according to different reaction temperatures, in the reaction conditions shown in Figure 6, to obtain verbenone with higher selectivity. The conversion of α-pinene was 57.5% when the reaction temperature was 55 °C and reached 83.2% at 85 °C, which indicated the significant effect of increasing the reaction temperature. The conversion changed less when the reaction temperature was further increased to 95 °C. This may be that the decomposition rate of TBHP at 95 °C does not increase much. While temperature and catalyst play an effect on the decomposition rate of TBHP, they also have a significant effect on the selectivity of α-pinene oxidation products. The selectivity of verbenone increased from 20.2% to 36.4% when the temperature was increased from 55 °C to 85 °C. When the temperature continued to increase to 95 °C, the selectivity of verbenone then decreased to 34.1%. This indicates that 85 °C is the optimal reaction temperature. The selectivity of verbenone increased from 0.9% to 3.6% when the temperature was increased from 55 °C to 95 °C. From the catalytic results obtained from different temperatures, it can be clearly concluded that the consumption of α-pinene and the production of verbenone and verbenol should be favored at a temperature of 85 °C.

#### 3.10.2. Effect of Reaction Time

In order to know the optimal reaction time for the higher selectivity synthesis of verbenone, experiments were carried out for different times using the conditions shown in Table 3. When the time was 6 h, the conversion of α-pinene and the selectivity of verbenone were 64.4% and 23.9%, respectively. The reaction was carried out under the same conditions for 12 h, and the conversion of α-pinene and the selectivity of verbenone increased significantly, reaching 84.7% and 40.1%, respectively. When the reaction time was 14 h, the growth of α-pinene conversion slowed down, the selectivity of verbenone did not increase, and it is possible that peroxide by-products started to be generated at this time. The selectivity of verbenone increased gradually during the increase in reaction time from 5 h to 10 h and, then, decreased with the increase in reaction time. It is possible that some of the verbenolides were oxidized to verbenone. Based on the catalytic activity of CuAPO-5 at different reaction times, it can be concluded that a reaction time of 12 h is favorable for the selectivity of verbenone.

#### 3.10.3. Effect of n(α-Pinene)/n(TBHP)

**Figure 7:**
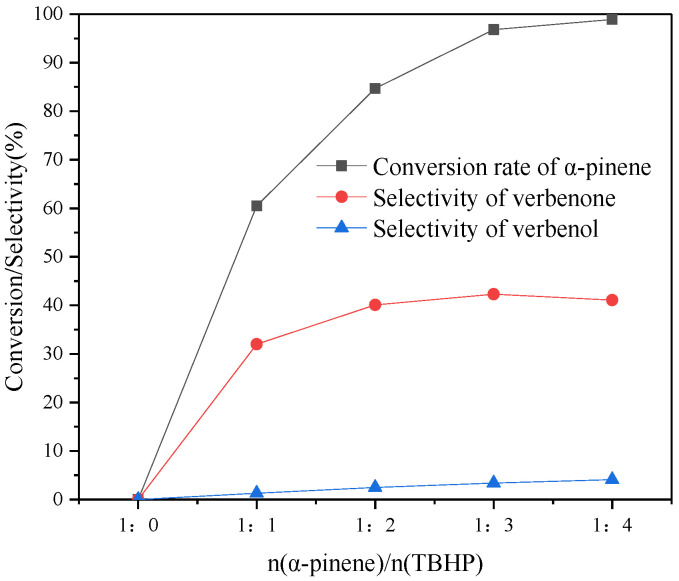
Variation of n(α-pinene):n(TBHP) in the oxidative synthesis of verbenone with TBHP. Reaction conditions: CuAPO-5(0.06) 100 mg, α-pinene 1 mmol, temperature 85 °C, TCM 10 mL, reaction 12 h.

In order to understand the effect of TBHP on the selectivity of verbenone, the oxidation reaction was carried out using different ratios of n(α-pinene)/n(TBHP), and the reaction conditions are shown in Figure 7. When no TBHP was added, the oxidation reaction of α-pinene did not occur. When n(α-pinene)/n(TBHP) = 1:3, the conversion of α-pinene and the selectivity of verbenone increased, substantially, to 96.8% and 42.3%, respectively. When the addition ratio of TBHP continued to increase, the conversion of α-pinene and the selectivity of verbenone changed less. It is possible that this is because α-pinene is basically completely converted under the condition of n(α-pinene)/n(TBHP) = 1:3. Based on the effects of different ratios on the reaction, the most suitable ratio was n(α-pinene)/n(TBHP) = 1:3.

#### 3.10.4. Effect of Solvent

The results of α-pinene oxidation occurring on CuAPO-5, under different solvent conditions, are shown in Table 4. It can be seen from the figure that the solvents caused a great influence on the activity of α-pinene. The conversion of α-pinene was 96.8%, 91%, and 85.4% in chloroform, acetonitrile, ethyl acetate, acetone, and DMF, respectively, while the conversion was only 25% and 13% in ethyl acetate and acetone. The selectivity of verbenone in different solvents also varied widely, and it was obvious that chloroform had the greatest effect on the selectivity of verbenone with 42.3% in the comparison of the solvents. The selectivity of verbenone was 38.7% and 33.1% when acetonitrile and DMF were used as solvents, respectively, while the selectivity of verbenone was 3.4%, 1.3%, and 9.2% when chloroform, acetonitrile, and DMF were used as solvents, respectively. Based on the effects of different solvents on the catalytic activity of CuAPO-5, it can be concluded that the solvent plays a key role in the oxidation of α-pinene, and chloroform should be selected as a suitable solvent. As can be seen from Table 4, except acetone and ethylacetate, other solvents contain elements other than C, H, and O. When acetone and ethylacetate were used as solvents, the conversion rate of α-pinene was lower, and chloroform, acetonitrile, and DMF were more effective. Among them, chloroform has the most significant effect on the reaction and plays a positive role in the oxidation of the allyl site. This may be because chloroform can promote the transfer of α-pinene in the two phases and promote the contact between α-pinene and TBHP, thus greatly improving the conversion of α-pinene.

#### 3.10.5. Effect of the Catalyst Amount

**Figure 8:**
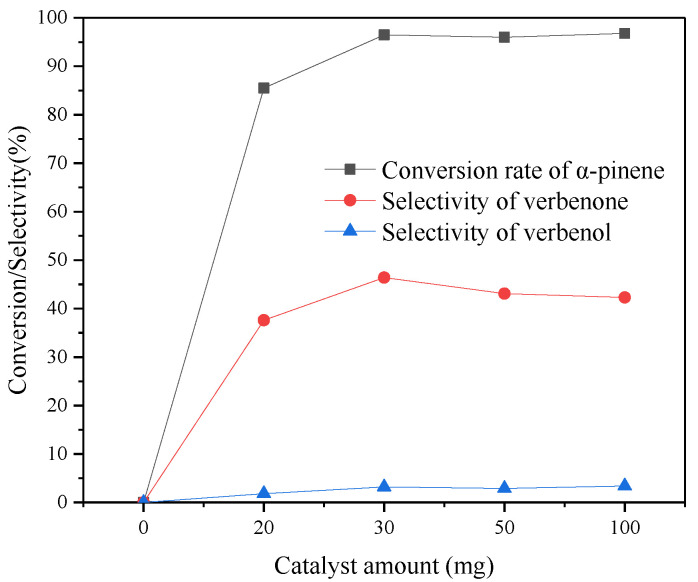
Effect of catalyst amount on the synthesis of verbenenone from α-pinene. Reaction conditions: α-pinene 1 mmol, 3 mmol of TBHP, temperature 85 °C, TCM 10 mL, reaction 12 h.

The influence of catalyst dosage on the synthesis of verbenone with α-pinene is shown in Figure 8. When no catalyst is added, α-pinene is not oxidized. When the dosage of CuAPO-5(0.06) increased from 20 mg to 100 mg, the conversion rate of α-pinene increased from 85.5% to 96.8%, and the selectivity of verbenone reached a maximum of 46.4% when the dosage reached 30 mg. Excessive catalyst is not conducive to the synthesis of verbenone.

#### 3.10.6. Recycling Efficiency of CuAPO-5

**Figure 9:**
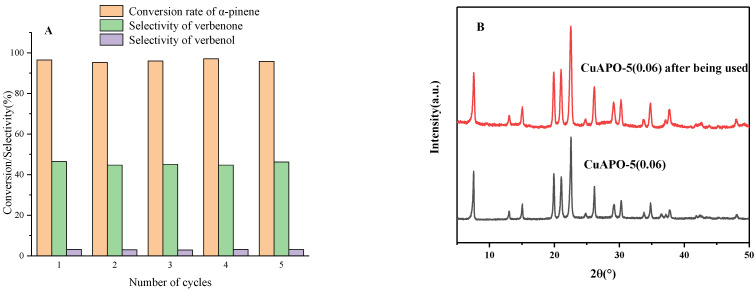
Stability check of CuAPO-5-catalyzed α-pinene synthesis of verbenone (**A**) and XRD spectrum of recovered CuAPO-5 (**B**).

The recycling efficiency of CuAPO-5 was checked for the selective oxidation of α-pinene to verbenone for five consecutive reaction cycles. The optimized reaction conditions were selected for the experiments. Additionally, 30 mg of CuAPO-5(0.06), 1 mmol of α-pinene, 3 mmol of 70% aqueous tert-butyl hydroperoxide, and 10 mL of chloroform were taken and reacted at 85°C for 12 h. After the reaction, the catalyst was washed with acetonitrile, dried at 150 °C, and then calcined at 550 °C for 2 h to proceed to the next cycle. In Figure 9A, we summarize the verbenone selectivity for five consecutive cycles, and it is clear, from these results, that the CuAPO-5 catalyst maintains its catalytic activity at all times. The catalysts exhibited almost identical activity over up to five reaction cycles. ICP-OES was used to analyze the elements of the recycled catalyst, and the copper content was 1.83 wt %. Compared with the fresh catalyst, there was no significant loss of copper content, indicating that copper can be well combined on the AlPO_4_-5 skeleton. The used CuAPO-5(0.06) molecular sieve was examined by X-ray diffraction, and the results are shown in Figure 9B. X-ray diffraction analysis of the catalyst after the reaction cycle showed that the CuAPO-5(0.06) structure was not destroyed. This result not only confirms the stability of the catalyst but also the recyclability of the same catalyst, over up to five reaction cycles, without loss of activity.

## 4. Discussion

Table 5 summarizes the important results published in the field of synthesis of verbenenone with α-pinene and the catalytic performance of the catalysts prepared in this study. According to previous literature, homogeneous catalysts often have more obvious catalytic advantages, but the use of homogeneous catalysts also brings environmental problems and inconvenient separation and recovery problems. The CuAPO-5 multiphase catalyst used in this work is simple to prepare, which reduces the cost of catalyst separation and recovery, and it may become a substitute for the synthetic catalyst used in the oxidation process in the future. In addition, the CuAPO-5 molecular sieve used in this work has a large specific surface area, which is conducive to the reaction on the surface of the catalyst. Through characterization analysis (combining XPS and EDS results), copper was uniformly dispersed on the molecular sieve in the form of divalence, which was conducive to the full contact between the reactant and the active site of the catalyst, thus promoting the reaction. When copper enters the skeleton, the low-priced metal replaces the high-priced element, resulting in excessive negative charge in the aluminum phosphate skeleton. In order to maintain the charge balance of the skeleton, it will bind protons and generate a Brownst acidic site. However, when the amount of copper introduced is too much, it is not conducive for copper to continue to enter the skeleton.

CuAPO-5 catalyzes the free radical reaction of α-pinene, which is easy to operate and conducive to industrial production. Among the tested catalysts, CuAPO-5(0.06) was found to have the highest catalytic activity, including the conversion rate of α-pinene (96.8%) and the selectivity of verbenenone (46.4%). This has a very positive impact on the exploration of the use of heterogeneous catalysts and the field of fine chemical engineering.

## 5. Conclusions

Based on our experimental results, we can conclude that CuAPO-5, with different Cu/Al ratios, was synthesized by a simple hydrothermal method. Using jade to fit the XRD spectra, the cell parameters increased with the appropriate addition of copper, indicating that Cu(II) can be successfully incorporated into the AFI aluminum phosphate skeleton. The characterization results showed that Cu(II) could be successfully incorporated into the aluminum phosphate backbone of AFI. Through the discussion of the catalytic reaction conditions, the optimal reaction conditions were obtained: 30 mg of CuAPO-5(0.06), 1 mmol of α-pinene, 3 mmol of TBHP, temperature 85 °C, TCM 10 mL, and a 12 h reaction. Under these conditions, CuAPO-5(0.06) showed good catalytic activity for the synthesis of verbenone by the oxidation of α-pinene. The conversion rate of α-pinene was 96.8%, and the selectivity of verbenone was 46.4%. No epoxidation of α-pinene was found in the products. This indicates that the catalyst prefers to attack the allyl than double bonds. After five cycles of use, its catalytic effect remained essentially unchanged. These results indicate that CuAPO-5 can selectively oxidize the allyl of organic compounds under environmentally friendly conditions.

## Figures and Tables

**Table 1:** Structural properties of AlPO_4_-5 and CuAPO-5 catalysts.

Catalyst	Surface Area, A_BET_ (m^2^·g^−1^)	Pore Volume, V_p_ (cm^3^·g^−1^)	Cu, wt%	Number of Acid Sites (mmol·g^−1^)			
				Weak ^1^	Moderate ^2^	Strong ^3^	Total Acid
AlPO_4_-5	359	0.44		0.10	0.09	0	0.19
CuAPO-5(0.03)	357	0.48	0.86	0.11	0.13	0.05	0.29
CuAPO-5(0.06)	359	0.48	1.88	0.12	0.14	0.05	0.31
CuAPO-5(0.09)	353	0.45	3.20	0.13	0.13	0.04	0.30

^1^ Weak acidity = ammonia desorption in about 170 °C. ^2^ Moderate acidity = ammonia desorption in about 255 °C. ^3^ Strong acidity = ammonia desorption in about 365 °C.

**Table 2:** Catalytic activity of α-pinene oxidation catalyzed by CuAPO-5 with different copper contents and TBHP-free experiments.

Entry	Catalysts	Conv. (%) α-Pinene	Selectivity (%)	
			Verbenol	Verbenone
1	Blank	0	0	0
2	CuAPO-5 (no TBHP)	0	0	0
3	CuAPO-5(0.03)	81.4	3.0	30.6
4	CuAPO-5(0.06)	83.2	3.5	36.4
5	CuAPO-5(0.09)	75.0	1.7	25.6

**Table 3:** Variation in the time of oxidative synthesis of verbenone by α-pinene and TBHP ^4^.

Entry	Time (h)	Conv. (%) α-Pinene	Selectivity (%)	
			Verbenol	Verbenone
1	6	64.4	2.7	23.9
2	8	75.2	3.2	31.8
3	10	83.2	3.5	36.4
4	12	84.7	2.5	40.1
5	14	87.5	1.9	39.2

^4^ Reaction conditions: CuAPO-5(0.06) 100 mg, α-pinene 1mmol, TBHP 2 mmol, temperature 85 °C, TCM 10 mL.

**Table 4:** Effects of different solvents on the synthesis of verbenenone from α-pinene ^5^.

Entry	Solvent	Conv. (%) α-Pinene	Selectivity (%)	
			Verbenol	Verbenone
1	Chloroform	96.8	3.4	42.3
2	Acetonirile	91.4	1.3	38.7
3	Acetone	13	0	3.1
4	Ethylacetate	25	2	5.4
5	DMF	85.4	9.2	33.1

^5^ Reaction conditions: 100 mg of CuAPO-5(0.06), 1 mmol of α-pinene, 3 mmol of TBHP, temperature 85 °C, 12 h reaction.

**Table 5:** Comparison of catalytic properties of different catalysts for the synthesis of α-pinene into verbenenone.

Entry	Catalyst	α-Pinene/Catalyst (mmol/mg)	Conditions	Solvent	Conv. (%) α-Pinene	Selectivity (%)	Ref.
						Verbenone	
1	AuCu/TiO_2_	5/100	TBHP (5 mmol), 24 h, 70 °C	acetonitrile	97	47.9	[38]
2	Ti-MCM-41	8.4/63	H_2_O_2_ (2.1 mmol), 7 h, 70 °C	acetonitrile	16.04	39	[39]
3	FePcCl_16_-NH_2_-SiO_2_	0.1875/8.4	TBHP (0.4875 mmol), 23 h, 40 °C	acetone	83.7	23	[40]
4	[VO(HMIMMPP)(H_2_O)]-Y	30/100	TBHP (75 mmol), 12 h, 80 °C	acetonitrile	45.28	40.69	[7]
5	CuFe_2_O_4_	1/100	TBHP (2 mmol), 20 h, 90 °C	acetonitrile	46.6	38.9	[18]
6	Neat [Fe(sal_2_bz)(H_2_O)_2_]_2_·2H_2_O	10/15	H_2_O_2_ (20 mmol), 24 h, 80 °C	acetonitrile	100	79	[41]
7	CuCl_2_	7.3/9.8	TBHP (44 mmol), 6 h, 70 °C	acetonitrile	100	78	[14]
8	CuAPO-5(0.06)	1/30	TBHP (3 mmol), 12 h, 85 °C	chloroform	96.8	46.4	This work ^6^

^6^ Reaction conditions: 30 mg of CuAPO-5(0.06), 1 mmol of α-pinene, 3 mmol of TBHP, temperature 85 °C, TCM 10 mL, 12 h reaction.
